# Spatial Representations Are Specific to Different Domains of Knowledge

**DOI:** 10.1371/journal.pone.0005543

**Published:** 2009-05-20

**Authors:** Rowena Beecham, Robert A. Reeve, Sarah J. Wilson

**Affiliations:** The Department of Psychology, School of Behavioural Science, University of Melbourne, Melbourne, Australia; Catholic University of Sacro Cuore, Italy

## Abstract

There is evidence that many abstract concepts are represented cognitively in a spatial format. However, it is unknown whether similar spatial processes are employed in different knowledge domains, or whether individuals exhibit similar spatial profiles within and across domains. This research investigated similarities in spatial representation in two knowledge domains – mathematics and music. Sixty-one adults completed analogous number magnitude and pitch discrimination tasks: the Spatial-Numerical Association of Response Codes and Spatial-Musical Association of Response Codes tasks. Subgroups of individuals with different response patterns were identified through cluster analyses. For both the mathematical and musical tasks, approximately half of the participants showed the expected spatial judgment effect when explicitly cued to focus on the spatial properties of the stimuli. Despite this, performances on the two tasks were largely independent. Consistent with previous research, the study provides evidence for the spatial representation of number and pitch in the majority of individuals. However, there was little evidence to support the claim that the same spatial representation processes underpin mathematical and musical judgments.

## Introduction

The cognitive representations of many abstract concepts (such as number, pitch, time) are putatively spatial in format [1]. For example, in many languages numbers are described in spatial terms (“low” or “high” numbers) [1]. Other examples include pitch (“low” or “high” tones), time (looking “forward” to future events), emotions (“high” spirits) and social relationships (“close” friends, “high” society) [1], [2]. More direct support for spatial representation in cognition comes from research demonstrating spatial stimulus-response compatibility effects (e.g., for number [3]–[9] and pitch [10]). Despite the suggestion of shared cognitive processes, to date most research has focused on cognitive processing within individual knowledge domains. Thus, the extent to which spatial representation processes are *shared* by different knowledge domains remains unclear. Moreover, because researchers tend to assume that all individuals possess similar cognitive systems, they use aggregate analytic methods to answer questions. Thus, it is unknown whether this organisation is typical of some or all individuals.

The domains of mathematics and music lend themselves to exploration of these issues. Anecdotal and experimental evidence suggests that there are similarities in the cognitive processes that underlie mathematical and musical competencies [11]–[16]. And, there is strong experimental evidence that both number and pitch are spatially represented in the cognitive system. In the number domain, the primary evidence is the Spatial-Numerical Association of Response Codes (SNARC) effect [3]. This effect reflects faster responses to number magnitude (explicit condition) or parity (implicit condition) judgements under “compatible” conditions (i.e., when responses to large numbers are on the right and small numbers are on the left) [3]–[9]. The parallel finding for pitch is the Spatial-Musical Association of Response Codes (SMARC) effect [10]. The SMARC task was designed as an analogue of the SNARC task and requires responses to pitch height (explicit condition) or timbre (implicit condition) judgements using keys near to (“lower”) or further from (“upper”) an individual's body. The SMARC effect reflects a faster response for “compatible” conditions (i.e., responding to lower pitches with the “lower” button and higher pitches with the “upper” button). Given the methodological similarities between the SNARC and SMARC tasks, comparing task performances allows investigation of whether spatial representation processes are shared across different knowledge domains.

Interestingly, observed small effect sizes and large variability estimates suggest substantial variability in performances on both SNARC and SMARC tasks [4]–[6], [10]. This might reflect individual differences in the organisation of the spatial representations of number and pitch. Surprisingly, scant attention has been paid to individual differences in cognitive architecture and there is growing concern that aggregating data across individuals may result in misleading claims [17]–[19]. Therefore, other methods that partition performance and identify subgroups with different response profiles appear warranted.

We used the SNARC and SMARC tasks to assess the spatial representation of number and pitch respectively. Given the variability in previous findings, we expected that subgroups of individuals with different SNARC or SMARC patterns would be identified. For individuals with similar response profiles on both tasks, a common spatial representational system underlying number and pitch would be suggested. Different patterns of performance might suggest that number and pitch utilise different representational systems.

## Results

### Identifying individual differences in performance

For each version (explicit and implicit) of the SNARC and SMARC tasks, clusters of individuals with different patterns of performance were identified using hierarchical cluster analyses with Ward's method of cluster linkage and Squared Euclidian distances Three clusters were characterized for each version of the tasks and exhibited one of the following response profiles:

1. SNARC/SMARC: responses were faster for the compatible than incompatible conditions.
2. Reverse SNARC/SMARC: responses were faster for the incompatible than compatible conditions.
3. Other: participants did not show either of the above patterns.

**Figure 1:**
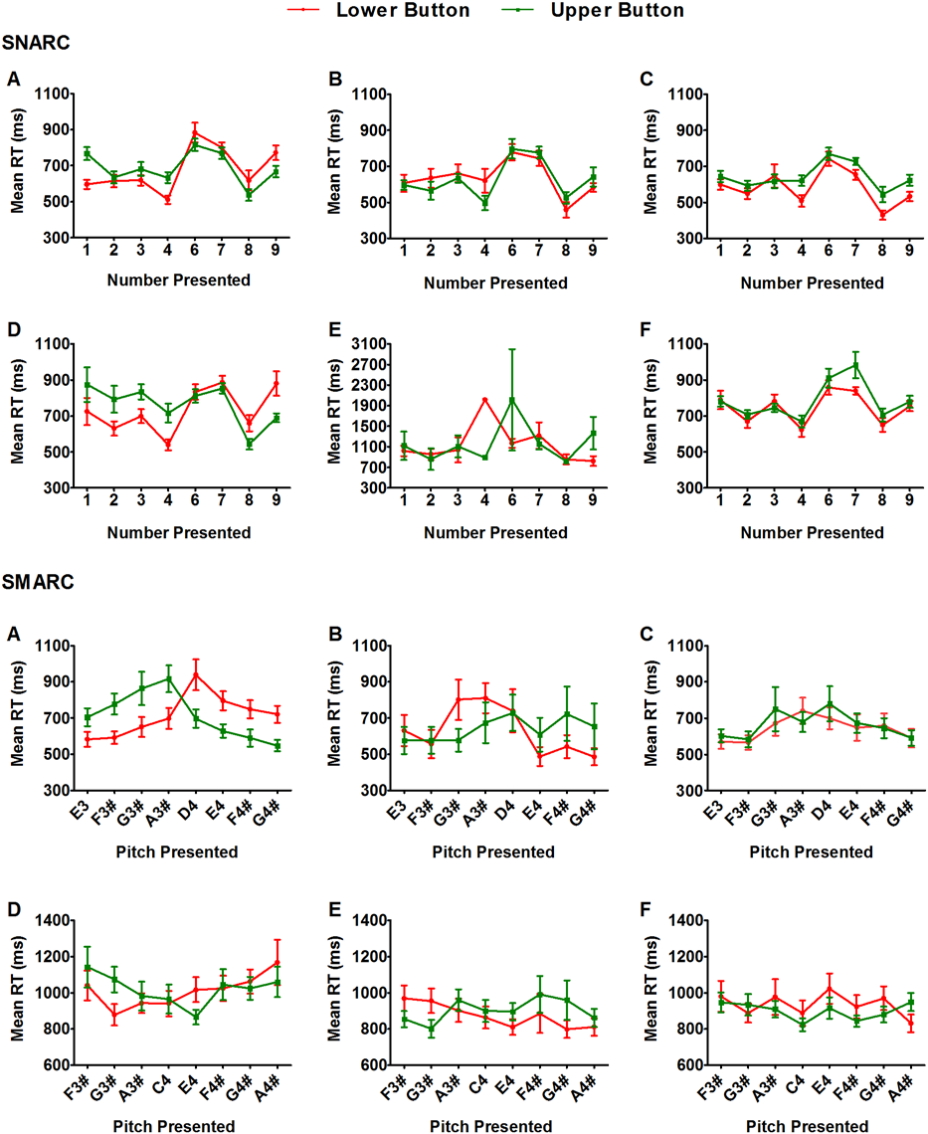
Mean reaction times and standard errors for each subgroup. (a) Explicit SNARC/SMARC subgroup; (b) Explicit Reverse SNARC/SMARC subgroup; (c) Explicit Other subgroup; (d) Implicit SNARC/SMARC subgroup; (e) Implicit Reverse SNARC/SMARC subgroup; (f) Implicit Other subgroup.

The response patterns of each subgroup are shown in Table 1 and Figure 1. To determine whether these profiles were statistically robust, repeated measures ANOVA with number magnitude/pitch height (low or high), compatibility, and distance (1, 2, 3, or 4 integers/tones from the centre of the range) as factors was performed for each subgroup. This form of analysis is consistent with previous research [3], [8], [10]. The results are presented separately for each response profile.

**Table 1:** Number of Participants and Average Difference between Compatible and Incompatible Response Times (ms) for Each Subgroup.

Version	Subgroup	n (%)	Average Difference^1^ (SD)
Explicit	SNARC	28 (46%)	81.5 (76.4)
	SMARC	29 (52%)	185.0 (148.9)
	Reverse SNARC	11 (18%)	−50.8 (41.9)
	Reverse SMARC	6 (11%)	−106.8 (81.4)
	Other (SNARC)	22 (36%)	−17.9 (66)
	Other (SMARC)	21 (37%)	3.3 (72.2)
Implicit	SNARC	22 (36%)	124.4 (96.8)
	SMARC	18 (35%)	79.7 (81.0)
	Reverse SNARC	2 (3%)	245.3 (172.6)
	Reverse SMARC	14 (27%)	−72.3 (61.3)
	Other (SNARC)	37 (61%)	−30.1 (84.6)
	Other (SMARC)	19 (37%)	4.9 (52.4)

1 Difference = incompatible response times minus compatible response times.

### SNARC/SMARC subgroups

Both the explicit and implicit SNARC subgroups demonstrated significant SNARC effects (explicit: *F*(1,26) = 33.13, *p*<.01; implicit: *F*(1,17) = 64.85, *p*<.01). These effects were more pronounced for smaller numbers (compatibility×magnitude: explicit: *F*(1,26) = 9.12, *p*<.01; implicit: *F*(1,17) = 19.90, *p*<.01), and, in the explicit condition, for the numbers one and nine (compatibility×distance: *F*(3,24) = 3.53, *p* = .03). Similarly, the SMARC subgroups demonstrated significant SMARC effects (explicit: *F*(1,28) = 63.51, *p*<.01; implicit: *F*(1,17) = 31.34, *p*<.01). In the implicit version, the SMARC effect was more evident for pitches further from the centre of the presented range (*F*(3,15) = 3.92, *p* = .03).

### Reverse SNARC/SMARC subgroups

The explicit Reverse SNARC subgroup showed a significant Reverse SNARC effect (*F*(1,10) = 29.95, *p*<.01). No ANOVA was conducted for the implicit Reverse SNARC subgroup due to the small number of participants (n = 2). Significant Reverse SMARC effects were seen for both Reverse SMARC subgroups (explicit: *F*(1,5) = 20.07, *p*<.01; implicit: *F*(1,13) = 22.66, *p*<.01). In the implicit version, the Reverse SMARC effect was more evident for pitches further from the centre of the presented range (*F*(3,11) = 4.82, *p* = .02) and for higher pitches (compatibility×pitch height: *F*(1,13) = 9.74, *p*<.01).

### Other subgroups

There were no significant differences between compatible and incompatible trials for the remaining subgroups (SNARC explicit: *F*(1,20) = 2.45, *p* = .13; SNARC implicit: *F*(1,33) = 0.73, *p* = .40; SMARC explicit: *F*(1,20) = 0.74, *p* = .40; SMARC implicit: *F*(1,18) = 0.59, *p* = .45), but left button responses were faster than right button responses for the SNARC Other subgroups (compatibility×magnitude: explicit: *F*(1,20) = 21.33, *p*<.01; implicit: *F*(1,33) = 9.82, *p*<.01).

### The influence of background variables on response profiles

Subgroup membership was cross-tabulated with mathematical or musical background and other demographic variables (such as age, gender or handedness). No significant relationships were found between SNARC subgroup membership and mathematical background or other demographic variables, or between SMARC subgroup membership and musical background or other demographic variables. That is, these variables did not distinguish between the subgroups for any condition. Furthermore, when participants were categorised as musicians or non-musicians according to criteria developed by Rusconi and colleagues [10], neither group demonstrated SMARC or Reverse SMARC effects.

### Overlaps in SNARC and SMARC task performances

Similarities between patterns of responses on the two tasks were investigated using Fisher's Exact Test. There were no significant relationships between subgroup memberships for the SNARC and SMARC tasks. This indicates that the response profiles of individuals on the SNARC task were unrelated to their response profiles on the SMARC task.

## Discussion

This study examined the extent to which spatial representation processes are shared by different knowledge domains, and whether this organization is typical of some or all individuals. Our analytic approach allowed us to identify several distinct spatial profiles underlying mathematical and musical judgments. However, despite using putatively analogous tasks with very similar response types, no overlap was seen between SNARC and SMARC task performances. That is, the profiles of individuals were different across domains.

These findings have broad methodological and conceptual implications. First, the identification of individuals with distinct response profiles challenges conventional wisdom of the homogeneity of cognitive architecture and encourages a re-evaluation of our approach to the study of human knowledge representation. The prevailing use of aggregate data analysis has masked individual differences in the spatial representation of number and pitch. The demonstration of subgroups with different response patterns likely accounts for the large variability and small differences between compatible and incompatible trials reported in previous SNARC and SMARC studies [4]–[6], [10]. By extension, our understanding of cognitive processes will be enhanced by the adoption of analytic methods that partition performance and identify subgroups with different response profiles.

Second, the minimal overlap between individual SNARC and SMARC profiles suggests that spatial representations used in different knowledge domains are largely separate, providing support for domain specific representational systems. It also raises important ontological questions about the evolution of cognitive representational systems. In particular, the parsimonious account of a cognitive system that shares processes between domains seems less likely than individuated systems that develop in response to specific environmental contexts. The similarities between individuated representational systems might arise because they are the most efficient or probable given the constraints of the human nervous system [20].

Finally, the findings extend our understanding of spatial processes. For each domain, most people (63–64%) utilized spatial representations of some form on explicit tasks, confirming the cognitive reality of spatial representations. However, the direction of numbers or pitches in these spatial representations appeared to vary between individuals, as suggested by the sizeable Reverse SNARC/SMARC subgroups. Of note, the weaker findings seen for the implicit conditions (e.g., smaller SNARC/SMARC subgroups) are likely attributable to: (a) the implicit task being a noisier task in which reaction times might also be influenced by parity or timbre; and (b) participants using implicit references that were not accounted for in the analyses.

The failure of one-third of the sample to demonstrate a spatial effect in either the SNARC or SMARC tasks when explicitly instructed to respond to number magnitude or pitch height is intriguing. Possible explanations include: (a) that these individuals do not spatially represent number magnitude or pitch height; (b) that the spatial representations of these individuals do not influence reaction times on SNARC or SMARC tasks. In other words, they might represent number or pitch in ways that do not easily map onto the position of the response keys; or (c) the format of the spatial representation may change according to task demands. This last conjecture is consistent with the previous demonstration of a classic SNARC effect when the schematic outline of a ruler was displayed (i.e., small numbers shown on the left and large numbers on the right) but the opposite pattern of associations when the schematic outline of a clock was displayed (i.e. small numbers shown on the right and large numbers on the left) [21]. Future studies incorporating a qualitative measure of each participant's subjective spatial representation would help address this issue.

The source of the observed inter-individual variability remains unclear. Although no relationships between patterns of performance and mathematical/musical background were detected in this study, the inclusion of more explicit measures of mathematical and musical abilities might reveal experience to be a factor underlying the observed variability between individuals. Biological factors such as gender might also underlie individual variation. Gender differences in performances on spatial tasks (e.g., a male advantage on spatial rotation tasks) have been well replicated [22]–[23] and it is possible that these extend to the spatial representations of number and pitch. While gender was not found to discriminate between subgroup memberships in the current research, the high proportion of females in the sample made it difficult to rigorously investigate gender differences in the SNARC and SMARC effects.

In conclusion, the current research indicates that many, but not all, individuals cognitively represent abstract concepts such as number or pitch in a spatial format. Despite this, spatial representation processes are not shared by different knowledge domains.

## Materials and Methods

### Ethics Statement

Written consent was obtained and the experiment was conducted with the approval of the Human Research Ethics Committee of the University of Melbourne.

### Participants

Sixty-one university students (nine males and 52 females) participated in the study. The median age band was 16–20 years. Participants had received an average of 12 years schooling in mathematics, and five years of musical training. All reported having normal hearing and normal or corrected-to-normal vision.

### Design and Measures

A within-subjects design was used to allow comparison of different task performances for each individual. Participants completed SNARC and SMARC tasks, each of which had explicit (responded to number magnitude or pitch height) and implicit (responded to number parity or tone timbre) conditions. The inclusion of an implicit version avoided confounding response labels (e.g., “high” and “low”) with the spatial properties of the stimulus. The order of task completion was counterbalanced across participants. In all tasks the independent variables were number magnitude/pitch height and the location of the correct response key. The dependent variables were reaction time and accuracy.

#### SNARC Task

The SNARC task involved the presentation of spoken number words (one to nine, but not five). Although SNARC task stimuli have typically been presented in the visual domain, auditory stimuli were used to make presentation of the items analogous to the SMARC task and to avoid confounding modality of stimulus presentation with domain. Participants also completed a ‘visual’ SNARC task that showed similar effects to the ‘auditory’ SNARC task, in keeping with previous research [8]. Stimuli were recorded as wav files on a Macintosh–9.2.2 system using commercial software (ProTools version 5.2.1) and a Shure SM58 microphone. Reaction time was measured from the onset of the stimulus. For each participant, mean reaction times for low numbers (1, 2, 3, and 4) and high numbers (6, 7, 8, and 9) were determined for each response button. Compatible trials occurred when the correct response to a low number was the left button and the correct response to a high number was the right button. A SNARC effect was considered evident when responses on compatible trials were faster on average than responses on incompatible trials.

#### SMARC Task

The SMARC task involved the presentation of tones of 1000 ms duration. Stimuli were wav files created using a Yamaha S80 keyboard. The tones were the same frequencies used by Rusconi and colleagues [10]. For the Explicit condition, the tones were sinusoidal waves of frequencies 165, 185, 208, 233, 294, 330, 370, and 415 Hz (corresponding to E3, F3#, G3#, A4#, D4, E4, F4#, and G4# respectively). There was also a reference tone of frequency 262 Hz, corresponding to C4. For the Implicit condition, the tones had frequencies 185, 208, 233, 261, 330, 370, 415, and 466 Hz; corresponding to F3#, G3#, A3#, C4, E4, F4#, G4#, and A4# respectively. All were normalised for amplitude. For each frequency there were six timbres, electronically generated to sound like different musical instruments. Two timbres were wind instruments (flute and clarinet), two were string instruments (violin and guitar), one was a brass instrument (French horn), and one was a percussive instrument (vibraphone). Pilot testing demonstrated that the wind and string instruments were easily recognised by the non-musicians. The brass and percussive instruments were included to replicate the method of Rusconi and colleagues [10]. In both conditions, reaction time was measured from the onset of the final tone. For each participant, mean reaction times for the lowest four pitches and highest four pitches were determined for each response button. Compatible trials occurred when the correct response to a low pitch was the ‘lower’ button and the correct response to a high pitch was the ‘upper’ button. A SMARC effect was considered evident when responses on compatible trials were faster on average than responses on incompatible trials.

### Apparatus

The experiment was run on an IBM-compatible Pentium 4 personal computer with an Intel 8280/DB/DBM/DA AC ’97 Audio Controller. Auditory stimuli were presented through Gamma LH085 dynamic headphones. Visual information was presented on a 17-inch Samsung colour monitor (model SyncMaster 753 s), with a refresh rate of 85 Hz and a spatial resolution of 1024×768 pixels. Responses were made via four buttons on a standard QWERTY keyboard, which were coloured with stickers (‘Q’-red, ‘P’-green, ‘6’-blue, and ‘space bar’-yellow). The presentation of stimuli and recording of responses was controlled by Inquisit software, version 2.0.

### Procedure

Participants were seated in a darkened room at an average of 55 cm from the monitor and wore headphones. For each task, instructions were displayed on the computer screen at the beginning of the task and prior to each block. All instructions referred to the buttons by their colour. Participants answered a number of demographic questions prior to completing the three experimental tasks.

For the SNARC task, participants were either informed that they would hear a spoken number word between one and nine. In the Implicit condition they responded to number parity. In the Explicit condition they responded to number magnitude. The response buttons were positioned to the left and right of the participant.

For the Implicit condition of the SMARC task, participants were informed that they would hear a tone and should respond to the type of instrument playing the tone. Some participants (n = 30) heard woodwind and string timbres while others (n = 31) heard brass and percussion timbres. For the Explicit condition, participants were informed that they would hear two consecutive tones and were to report whether the second tone was higher or lower in pitch than the first. The response buttons were positioned near and far from the participant.

Participants completed 64 experimental trials (4 blocks of 16 trials) for each task. The first two blocks for each task comprised Implicit trials, and the last two blocks comprised Explicit trials. This ensured that participants completing the Implicit trials had not received any explicit cues to attend to the number magnitude/pitch height of the stimuli that might bias responding in the Implicit trials. Within each condition (Implicit/Explicit), low/odd numbers or low/woodwind/percussion tones were paired with the left/‘lower’ response key for one block and with the right/‘upper’ response key for one block.

The procedure for each block was the same for all tasks. Each block comprised two practice trials that were randomly selected from the range of possible trials for that block; and 16 experimental trials. The number or pitch presented on each experimental trial was randomised with the caveat that each number or pitch was presented twice in each block. In the Implicit SMARC blocks, of the two presentations of each pitch, one was a woodwind/brass timbre and the other was a string/percussive timbre. Participants were invited to take a break between each block, and could start the next block when ready by pressing the space bar.

For all tasks, each trial began with the presentation of a black fixation point (+) that remained for 250 ms. The numbers/tones were then presented. For the Explicit condition of the SMARC task, there was an interval of 250 ms between the two tones. Each trial ended when a response was given or 5000 ms after the onset of the final stimulus. Feedback was given following each trial, in the form of the words “correct” or “incorrect” appearing in the centre of the screen for 750 ms. There was an inter-trial interval of 1500 ms.
